# Heparan Sulfate Facilitates Spike Protein-Mediated SARS-CoV-2 Host Cell Invasion and Contributes to Increased Infection of SARS-CoV-2 G614 Mutant and in Lung Cancer

**DOI:** 10.3389/fmolb.2021.649575

**Published:** 2021-06-11

**Authors:** Jingwen Yue, Weihua Jin, Hua Yang, John Faulkner, Xuehong Song, Hong Qiu, Michael Teng, Parastoo Azadi, Fuming Zhang, Robert J. Linhardt, Lianchun Wang

**Affiliations:** ^1^Department of Molecular Pharmacology and Physiology, Morsani College of Medicine, University of South Florida Health, Tampa, FL, United States; ^2^Complex Carbohydrate Research Center, University of Georgia, Athens, GA, United States; ^3^Department of Biochemistry and Molecular Biology, University of Georgia, Athens, GA, United States; ^4^Department of Chemistry and Chemical Biology, Center for Biotechnology and Interdisciplinary Studies, Rensselaer Polytechnic Institute, Troy, NY, United States; ^5^Department of Chemical and Biological Engineering, Center for Biotechnology and Interdisciplinary Studies, Rensselaer Polytechnic Institute, Troy, NY, United States; ^6^Department of Biomedical Engineering, Center for Biotechnology and Interdisciplinary Studies, Rensselaer Polytechnic Institute, Troy, NY, United States; ^7^Shanghai Institute of Materia Medica, Chinese Academy of Sciences, Shanghai, China; ^8^Division of Allergy and Immunology, Department of Internal Medicine, Morsani College of Medicine, University of South Florida, Tampa, FL, United States

**Keywords:** heparan sulfate, SARS-Cov-2, spike protein, G614 mutant, lung cancer

## Abstract

The severe acute respiratory syndrome (SARS)-like coronavirus disease (COVID-19) is caused by SARS-CoV-2 and has been a serious threat to global public health with limited treatment. Cellular heparan sulfate (HS) has been found to bind SARS-CoV-2 spike protein (SV2-S) and co-operate with cell surface receptor angiotensin-converting enzyme 2 (ACE2) to mediate SARS-CoV-2 infection of host cells. In this study, we determined that host cell surface SV2-S binding depends on and correlates with host cell surface HS expression. This binding is required for SARS-Cov-2 virus to infect host cells and can be blocked by heparin lyase, HS antagonist surfen, heparin, and heparin derivatives. The binding of heparin/HS to SV2-S is mainly determined by its overall sulfation with potential, minor contribution of specific SV2-S binding motifs. The higher binding affinity of SV2-S G614 mutant to heparin and upregulated HS expression may be one of the mechanisms underlying the higher infectivity of the SARS-CoV-2 G614 variant and the high vulnerability of lung cancer patients to SARS-CoV-2 infection, respectively. The higher host cell infection by SARS-CoV-2 G614 variant pseudovirus and the increased infection caused by upregulated HS expression both can be effectively blocked by heparin lyase and heparin, and possibly surfen and heparin derivatives too. Our findings support blocking HS-SV2-S interaction may provide one addition to achieve effective prevention and/treatment of COVID-19.

## Introduction

The recent emergence of the novel, pathogenic Severe Acute Respiratory Syndrome Coronavirus 2 (SARS-CoV-2) worldwide poses a global health emergency (Graham and Baric, 2020). To develop specific anti-coronavirus therapeutics and prophylactics, the molecular mechanisms that underlie viral infection need firstly to be defined. The spike protein of coronaviruses facilitates viral entry into target cells including the initial binding on target cell surface and the following fusion of viral and cellular membrane. Pathogenesis studies have illuminated that the spike protein of SARS-CoV-2 (SV2-S) binds to angiotensin-converting enzyme 2 (ACE2) on host cell surface for cell entry, and then is primed by proteinases, including transmembrane serine protease 2 and cathepsin B and L, to mediate viral and host cell membrane fusion (Hoffmann et al., 2020; Walls et al., 2020a). It remains incompletely understood the molecular mechanisms by which SV2-S mediates SARS-CoV-2 infection of host cells (Hoffmann et al., 2020; Walls et al., 2020a).

Heparan sulfate (HS) is a linear polysaccharide that is ubiquitously expressed by most cell types in mammals at the cell surfaces and in the extracellular matrix. HS is used by multiple viruses to attach to the host cell surface. Mechanistically, viruses exploit the HS interaction to increase their concentration at the cell surface and augment their chances of finding a more specific entry receptor (Rusnati et al., 2009; Aquino and Park, 2016; Cagno et al., 2019). Human coronaviruses NL63 and OC43 bind to cell surface HS (de Haan et al., 2008; Milewska et al., 2014). Human coronavirus NL63 utilizes HS for attachment to target cells and employs ACE2 for following host cell entry (Milewska et al., 2014). The recent studies, including our own, reported that SV2-S binds heparin, a highly sulfated form of HS, and chemically synthesized HS (Clausen et al., 2020; Kim et al., 2020; Liu et al., 2020; Tandon et al., 2020; Tiwari et al., 2020a; Zhang et al., 2020c). These studies also showed that heparin inhibits SV2-S pseudotyped virus infection of Vero cells (kidney epithelial cell). Recent studies further reported that HS is essential for SV2-S-mediated SARS-CoV-2 cell entry; however, the correlation of HS expression with cell surface SV2-S binding and the required sulfation structure feature within cell surface HS have not been fully examined (Clausen et al., 2020). Meanwhile, the SARS-CoV-2-S G614 mutant has predominated globally (Walls et al., 2020b; Zhou et al., 2020) and several retrospective cohort studies highlighted that cancer patients are at increased risk for COVID-19 severity and fatality (Liang et al., 2020; Mehta et al., 2020; Poortmans et al., 2020; Rogado et al., 2020; Yu et al., 2020; Zhang et al., 2020a, b), while the underlying molecular mechanisms remain largely unknown.

In this study, we determined that SV2-S binding depends on and correlates with host cell surface HS expression. This binding is required for SARS-Cov-2 virus to infect host cells and can be blocked by heparin lyase, HS antagonist surfen, heparin, and heparin derivatives. The binding of heparin/HS to SV2-S is mainly determined by its overall sulfation with potential, minor contributions of specific SV2-S binding motifs. The higher binding affinity of SV2-S G614 mutant to heparin and upregulated HS expression on the cell surface may be one of the mechanisms underlying the higher infectivity of the SARS-CoV-2 G614 variant and the high vulnerability of lung cancer patients to SARS-CoV-2 infection, respectively. The higher host cell infection by SARS-CoV-2 G614 variant pseudovirus and the increased infection caused by upregulated HS expression both can be effectively blocked by heparin lyase and heparin, and possibly surfen and heparin derivatives too.

## Materials and Methods

### Cells

The cell lines A549, SH-SY5Y, 293T, THP-1 were obtained from the American-Type Culture Collection (ATCC, Manassas, VA, United States) (Table 1). The cell lines H441 and Calu-3 were purchased from Thermo Fisher Scientific (Waltham, MA, United States). The primary human lung microvascular endothelial cells (HMVECs) were obtained from Lonza (Basel, Switzerland). The mouse lung endothelial cell (MLEC) lines were generated in our lab (Wang et al., 2005; Wijelath et al., 2010; Qiu et al., 2013, 2018; Zhang B. et al., 2014). The cells were cultured in the conditions recommended by the vendors or as reported (Table 1).

**TABLE 1:** Summary of cells and their culture condition.

Name	Cell type	Source	Culture condition
PNT2	Normal human prostate epithelium immortalized with SV40	Millipore Sigma	RMPI 1640, 2 mM Glutamine, 10% FBS
SH-SY5Y	Human neuroblastoma cell line	ATCC	DMEM/F-12, 10% FBS
H441	Human lung adenocarcinoma epithelial cell	Thermo Fisher Scientific	RMPI 1640, 2 mM Glutamine, 10% FBS
HLMVEC	Human lung microvascular endothelial cells	Lonza	EGM-2 MV bullet kit
MLEC	Mouse lung endothelial cells	Generated in own lab	DMEM, 10% FBS
A549	Human adenocarcinoma alveolar basal epithelial cells	ATCC	RMPI 1640, 2 mM Glutamine, 10% FBS
293T	Human embryonic kidney 293 cells immortalized with SV40	ATCC	DMEM, 10% FBS
mTHP-1	Human macrophage induced from THP-1	ATCC	RPMI-1640, 2 mM Glutamine, 10% FBS 100 ng/mL PMA
Calu-3	Human lung adenocarcinoma epithelial cell	AddexBio	AddexBio-formulated EMEM, 10% FBS

### HS, Heparin, and Chemically Modified Heparins

The porcine intestinal heparin (16 kDa) and HS (11.7kDa) were obtained from Celsus Laboratories, Cincinnati, OH, United States. *N*-desulfated/*N*-acetylated heparin (NDS-HP,14kDa) and oversulfated heparin (OS-HP, 16 kDa) were obtained from Glycomed Inc. (Alameda, CA, United States). 2-*O*-desulfated heparin (2DS-HP, 13 kDa) and 6-*O*-desulfated heparin (6DS-HP, 13 kDa) were prepared as we reported previously (Wang et al., 2002; Bobardt et al., 2004; Zhang et al., 2012, 2013; Zhang F. et al., 2014). All the chemically-modified heparins have no anticoagulant activity as determined by amidolytic anti-factor Xa assay and were negative for endotoxin in the Limulus test (Wang et al., 2002).

### Flow Cytometry

Cells were seeded in collagen I coated cell culture dish. Confluent cells were treated with or without heparinase I, II, and III (HSase, 5 mU/ml of each) in DPBS at 37°C for 30 min and followed by adding collagenase IV for additional 30 min incubation at 37°C. Then cells were detached and suspended in DPBS-1% BSA for cell surface SV2-S binding or expression of HS or ACE2 analysis. SV2-S binding on cell surface was measured by incubating cells with 250 nM His-tagged SV2-S (Sino Biological, 40591-V08B1). Cell surface-bound SV2-S was measured by flow cytometry (Canto II 488 Laser) after staining with Alexa Fluor488-conjugated goat anti-His antibody. Cell surface HS level was examined after sequential staining with anti-HS antibody 10E4 (Amsbio, 370255-1) at 2.5 μg/ml and Alexa Fluor488 goat anti-mouse IgM secondary antibody. Cell surface ACE2 level was determined after staining with anti-ACE-2 antibody (R&D Systems, AF933) at 1 μg/ml and Alexa Fluor 488 donkey anti-goat IgG secondary antibody. Dead cells were gated based on DAPI staining. The collected data were analyzed using Prism 8 (GraphPad Software). Relative median fluorescence intensity (MFI) was calculated by subtracting MFI of isotype control from antigen staining or BSA from SV2-S.

### Cell-Based Enzyme-Linked Immunosorbent Assay (ELISA)

HS-deficient MLEC were seeded at 4 × 10^4^ cells per well in 96 well plates. After overnight culture, the cells were washed and fixed in 4% paraformaldehyde at room temperature (RT) for 15 min. After washing and blocking in 5% BSA overnight at 4°C, 20 nM His-tagged SV2-S was applied and incubated for 1 h at RT. Then cells were washed and incubated with anti-His secondary antibody conjugated with HRP for 1 h at RT. After intensive washing, ELISA substrate was applied. After 25 min, the color development was stopped with 0.5 M HCl. Absorbance at 450 nm was measured. Cells were further stained with Janus Green (Abcam, ab111622) for cell density. Absorbance at 595 nm was measured representing cell density and used to normalize cell surface SV2-S binding.

### Inhibitory Effect of Heparin and Chemically-Modified Heparins on Cell Surface SV2-S Binding

Cell-based ELISA protocol described above was used to determine inhibition. A549 cells were incubated with 20 nM His-tagged SV2-S in the absence or presence of different concentrations of heparin or chemically modified heparin. The binding was normalized to wells without the test compound to determine inhibitory effect.

### Interaction of SV2-S With Immobilized Heparin in Surface Plasmon Resonance (SPR) Analysis

Surface plasmon resonance analyses were performed using BIAcore 3000 (GE Healthcare, Uppsala, Sweden) to measure the binding kinetics and interaction affinity of SV2-S to heparin as we did previously (Condac et al., 2012; Zhang et al., 2013; Zhang B. et al., 2014). Biotinylated heparin was immobilized on streptavidin-coated Sensor SA chips (Zhang et al., 2013; Zhang B. et al., 2014; Zhang F. et al., 2014). For direct binding analysis, SV2-S D614 (Sino Biological, 40591-V08H) and SV2-S G614 (Sino Biological, 40591-V08H3) were diluted in a running HBS-EP buffer containing 0.01 M HEPES, 0.15 M NaCl, 3 mM EDTA, 0.005% surfactant P20, at pH 7.4. Different concentrations of the proteins were injected over the heparin chip at a flow rate of 30 μl/min. At the end of the injection, a buffer was flowed over the sensor surface to allow dissociation. After 3 min, the sensor surface was regenerated by injecting 30 μl of 2 M NaCl. The response was monitored as a function of time (sensorgram) at 25°C. The sensorgrams of various SV2-S concentrations were fit globally to obtain association rate constant (*k*_*a*_), dissociation rate constant (*k*_*d*_), and equilibrium dissociation constant (*K*_*D*_) using the BiaEvaluation software version 4.0.1 (GE Healthcare, Uppsala, Sweden) and assuming a 1:1 Langmuir model.

### Transient and Stable Expression of ACE2 in 293T Cells

293T-ACE2-GFP expression cell lines were established by transfecting 293T cells with an ACE2-GFP expression vector (OriGene Technologies, RG208442). Two days after transfection, transient 293T-ACE-GFP cells were used for assessing SV2-S binding by flow cytometry. After transfections, GFP positive cells were sorted and seeded at one cell per well of 96 well plates. The transfected 293T cell clones with stable GFP and ACE2 expression were used for the pseudotyped virus infection assay.

### Production of SARS-CoV-2 Pseudotyped Virus

The following plasmids were obtained from Addgene including pcDNA3.1-SARS2-Spike mammalian expression plasmid (#145032), NL4-3 mCherry Luciferase dual reporter vector (#44965), and the control env expression vector, VSV-G (pMD2.G) (#12259). The SARS-CoV-2 G614 mutant expression vector was generated as recently reported (Zhang et al., 2020a). The SARS-CoV-2 pseudotyped virus was produced by transfecting 293T cells with 10 μg of NL4-3 mCherry Luciferase and 10 μg env expression vector: either VSV-G (pMD2.G) or pcDNA3.1-SARS2 Spike using polyethylenimine (PEI) (25 kDa, 1 μg/μL). The virus supernatant was collected at 48 h after transfection and concentrated at 1:100 ratio using Lenti-X^TM^ Concentrator (TaKaRa/Clontech, 631231). Viral titer was determined using Lenti-X^TM^ qRT-PCR Titration Kit (TaKaRa/Clontech, 631235).

### Pseudotyped Virus Infection Assay

293T-ACE2 cells were seeded at 1.25 × 10^4^ per well in poly-L-Lysine coated white 96 well plate and cultured overnight. Following, the wells were washed with DMEM and incubated with SARS-CoV-2-S pseudotyped virus and positive control VSV pseudotyped virus. At 6 h post-infection, the medium was changed with fresh DMEM supplemented with 10% FBS. At 48 h post-infection, the cells were lysed and measured for luciferase activity using a luciferase assay kit (Promega, E1500).

### Inhibition of SAS-Cov-2 Pseudotyped Virus Infection Assay

Pseudotyped virus infection assay was performed as described above. Before adding the pseudotyped virus into the well, cells were pre-incubated with DMEM supplemented with HSase, surfen (Sigma, S6951), HS, heparin, NDS-HP, 6DS-HP, 2DS-HP, or OS-HP for 30min at 37°C.

### *Ext1* Overexpression in 293T-ACE2 Cell Line

We used a human *Ext1* expression lentiviral vector (pLenti-GIII-CMV-GFP-2A-Puro based), which was obtained from abmgood (#195860610395) to overexpress *Ext1*. For lentivirus production, the *Ext1* expression vector (10 μg) and the two packaging viral vectors, pMD.2G (5 μg) and psPax2 (5 μg), both of which were obtained from Addgene, were co-transfected into 293T cells using PEI. The virus supernatant was collected at 48 h after transfection and concentrated at 1:100 ratio using a Lenti-X^TM^ Concentrator kit. To overexpress *Ext1*, 293T-ACE2 cells were inoculated with 1:100 diluted *Ext1* overexpression lentivirus with 8 μg/ml polybrene (Santa Cruz Biotech, sc-134220). Two days post lentivirus infection, cells were used for the experiment and *Ext1* overexpression efficiency was assessed by RT-qPCR.

### Reverse Transcription-Quantitative PCR (RT-qPCR)

RT-qPCR was performed to analyze the mRNA expression levels of *Ext1*. TRIzol reagent (Invitrogen) was used to extract the RNA from the 293T-ACE2 cells and then reverse transcribed into cDNA using the Bio-Rad reverse transcription system (#1708840). Bio-Rad SYBR-Green Supermix (#1708880) was used for qPCR analysis. The primer sequences used in this experiment were: *Ext1* forward, 5′-GCTCTTGTCTCGCCCTTTTGT-3 and reverse, 5′-GGTGCAAGCCATTCCTACC-3′; *glyceraldehyde 3-phosphate dehydrogenase* (*GAPDH*) forward, 5′-GTATTGGGCGCCTGGTCACC-3′ and reverse, 5′-CGGGAAGATGGTGATGG-3′. The PCR-amplified mRNA was quantified and the results were normalized against *GAPDH* expression. The 2^–ΔΔ^*^*C*^*^*q*^ method was used to calculate the relative mRNA expression level.

### Analysis of HS Gene and *ACE2* Expression in Lung Squamous Cell Carcinoma Patients and Health Control

Valuable cancer-related RNA sequencing data deposited in The Cancer Genome Atlas (TCGA) has provided ample opportunities for data mining and a deeper understanding of gene functions in cancer (Cancer Genome Atlas Research et al., 2013). For this purpose, the Gene Expression Profiling Interactive Analysis 2 (GEPIA2) software was developed and has aided the investigation of various genes in multiple cancer types leading to identification of potential biomarkers and therapeutic targets (Liang et al., 2018; Cai et al., 2019; Tang et al., 2019). To determine if HS gene and *ACE2* expressions are dysregulated in lung squamous cell carcinoma (LUSC), using the GEPIA2 software, the data deposited in TCGA including 486 LUSC patients with 50 normal controls were analyzed for their differential expression and relative expression abundance. The differential gene expression among LUSC pathological stage I-IV was analyzed also. The HS gene analyzed included *Ext1-2*, *Ndst1-4*, *Hs2st*, *Hs6st1-3*, *Hs3st1-6*, and *Sul1-2* (Qiu et al., 2018).

### Statistical Analysis

Statistical analysis was carried out with Prism 8 for Macintosh. All data are presented as mean ± SD or mean ± SEM and analyzed using a student’s *t*-test for two-group comparison. The two-parameter correlation was determined using the e Pearson’s correlation coefficient analysis. In all tests, *P*-value < 0.05 was considered statistically significant.

## Results

### Cell Surface SV2-S Binding Depends on and Correlates With HS Expression

**FIGURE 1:**
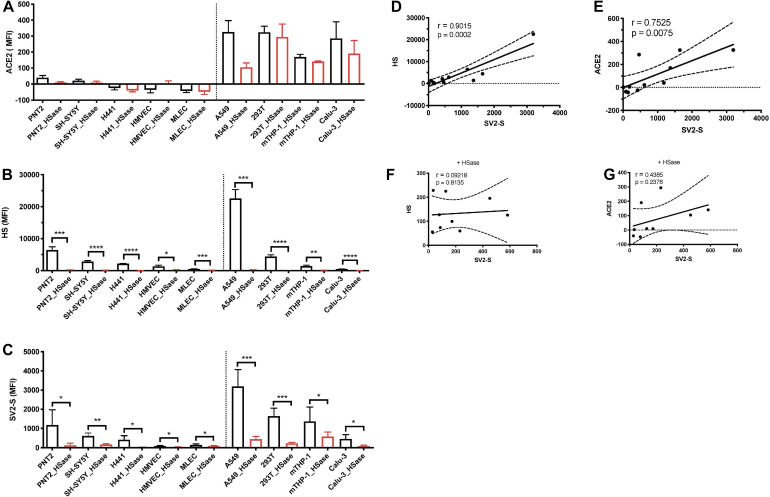
Cell surface SV2-S binding depends on and correlates with expression levels of HS. **(A,B)** Cell surface expressions of ACE2 and HS were assessed by flow cytometry after incubation with anti-ACE2 and anti-HS antibody 10E4, respectively, and sequential Alexa Fluor 488-tagged secondary antibody. A portion of the cells was treated with heparinases I-III (HSase) before the antibody staining. **(C)** The binding of SV2-S on cell surface was assessed by flow cytometry after sequential incubation with his-tagged SV2-S and Alexa Fluor 488-tagged anti-his antibody. A portion of the cells was treated with HSase before SV2-S binding. Median fluorescence intensity (MFI) was calculated by subtracting MFI of BSA binding or isotype-match naive control antibody binding. The experiments were repeated 3–6 times and an unpaired *t*-test was performed for comparison of the same cells without vs. with HSase treatment. Data are presented as mean ± SD. *p < 0.05, **p < 0.01, ***p < 0.001, ****p < 0.0001. **(D–G)** The Pearson’s correlation coefficient between SV2-S binding and cell surface expression level of HS or ACE2 was determined by analyzing the data collected in **(A–C)** using Prism 8. The cells analyzed in **(F,G)** were pre-treated with HSase.

Reported studies have determined that HS is required for cell surface SV2-S binding, but it remains unclear if the binding is cell-context dependent including ACE2 co-expression (Clausen et al., 2020). To address this issue, we analyzed available cell lines and primary cells available in our lab, including PNT2 (normal human prostate epithelial), SH-SY5Y (human neuroblastoma), H441 and Calu-3 (human lung adenocarcinomas), primary HMVEC, MLEC lines, A549 (human adenocarcinoma alveolar basal epithelial), 293T (human embryonic kidney), and mTHP-1 (macrophage differentiated from THP-1, a human acute monocytic leukemia cell line, by phorbol 12-myristate-13-acetate) (Table 1). Among these cells, A549, 293T, mTHP-1, and Calu-3 also express low levels of ACE2 on cell surface (Figure 1A). These cells express different levels of HS on cell surface assessed by anti-HS antibody 10E4 staining followed by flow cytometry analysis (Figure 1B). Cell surface binding of recombinant SV2-S of these cell lines was assessed by flow cytometry analysis (Figure 1C). The cell surface SV2-S binding positively correlated with the cell surface HS expression with a Pearson’s correlation coefficient of 0.9015 (Figure 1D) and with cell surface ACE2 expression of a Pearson’s correlation coefficient of 0.7515 (Figure 1E). Treating the cells with HSase, which diminished cell surface HS, did not affect ACE2 expression (Figures 1A,B). The HSase treatment diminished cell surface SV2-S binding and the positive correlation of cell surface ACE2 expression with SV2-S binding (Figures 1C,F,G), showing that SV2-S binding is determined by cell surface HS even if endogenous ACE2 is expressed on cell surface.

### HS Determines Cell Surface SV2-S Binding Only When ACE2 Is Expressed at Low Levels

**FIGURE 2:**
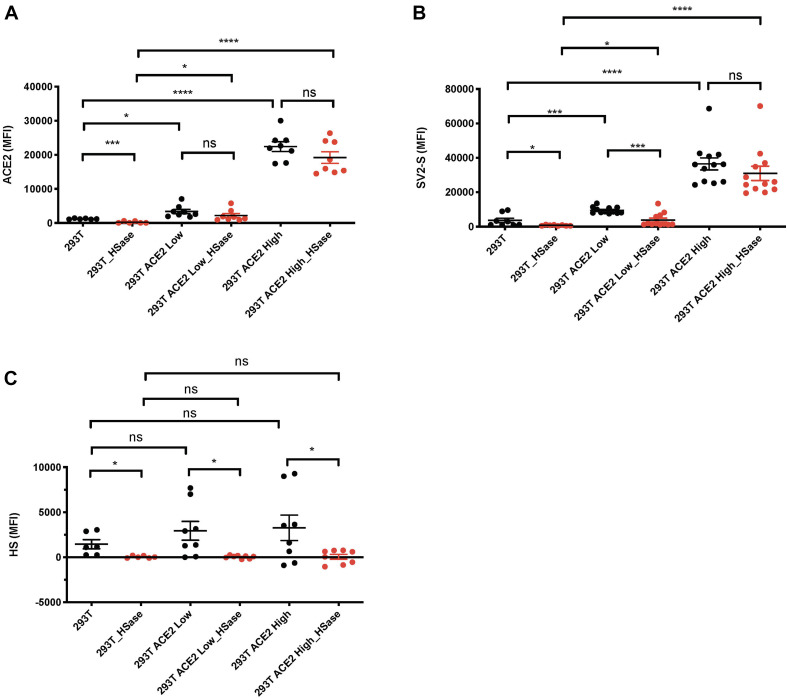
Heparan sulfate (HS) plays a major role in mediating cell surface SV2-S protein binding only when ACE2 expresses at a low level. 293T cells were transfected with ACE2-GFP or scramble-GFP plasmid. In SV2-S binding analysis, the transient 293T-ACE2-GFP-expressing cells were gated into two populations: the ACE2-GFP-low and -high expression cells based on GFP expression. **(A)** Cell surface ACE2 expression was assessed by flow cytometry after staining with anti-ACE2 antibody and Alexa Fluor 647-conjugated secondary antibody. A portion of the cells was treated with HSase before the specific antibody staining. **(B)** The binding of SV2-S on cell surface was assessed by flow cytometry after sequential incubation with his-tagged SV2-S and Alexa Fluor 647-tagged anti-his antibody. A portion of the cells was treated with HSase before SV2-S binding. **(C)** Cell surface HS expression was assessed by flow cytometry after staining with anti-HS antibody and Alexa Fluor 647-conjugated secondary antibody. A portion of the cells was treated with HSase before the specific antibody staining. MFI was calculated by subtracting MFI of BSA binding or isotype-match naive control antibody staining. The experiments were repeated 6- to 12- times and an unpaired *t*-test was performed for comparison of the same cells without vs. with HSase treatment. Data are presented as mean ± SD. ns, not significant; *p < 0.05, ***p < 0.001, ****p < 0.0001.

We observed that cell surface SV2-S binding was determined by HS, but this occurred in the absence or presence of low levels of endogenous ACE2 expression. Since SV2-S has a high binding affinity to ACE2 (Lu and Sun, 2020), we suspected that HS dependence would be attenuated when ACE2 expression is high. To test this idea, 293T cells were transfected with an ACE2-GFP expression plasmid, and the transient 293T-ACE2-GFP-expressing cells were gated into two populations: the ACE2-GFP low and high expression cells based on GFP expression. Anti-ACE2 antibody staining confirmed that the ACE2-GFP low and -high expression cells express low- and high ACE2 on cell surface, respectively (Figure 2A). The transiently-transfected 293T-ACE2-GFP cells showed increased cell surface SV2-S binding, positively correlating with their cell surface ACE2 expression (Figure 2B). Treating the cells with HSase diminished cell surface HS (Figure 2C) and SV2-S binding on the low ACE2-expressing 293T cells (Figure 2B). The HSase treatment did not significantly reduce cell surface SV2-S binding on the high ACE2-expressing 293T cells (Figure 2B). These data show that cell surface HS plays a determining role in mediating cell surface SV2-S binding only when ACE2 expression is low or absent.

### The *N*- and 6-*O*-Sulfation Are the Major Modifications Required for Heparin to Bind SV2-S and the Binding Also Depends on Overall Sulfation

**FIGURE 3:**
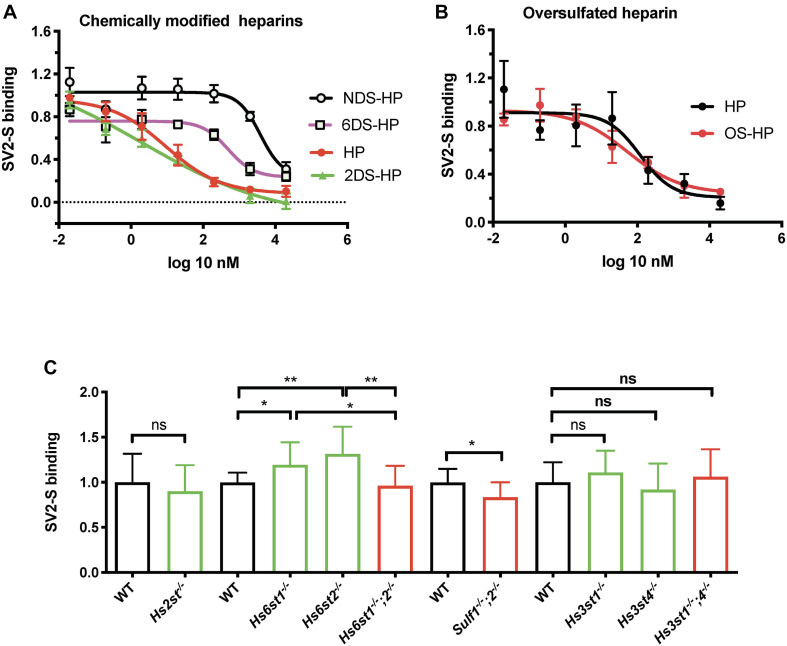
The modification of heparin and cell surface HS involved in SV2-S binding. **(A,B)** Inhibition on the binding of SV2-S to cultured A549 cells. After incubation in the absence or presence of different concentrations of heparin (HP), NDS-HP; 2DS-HP, 6DS-HP, or OS-HP, SV2-S that bound to A549 cells was quantified by cell-based ELISA and normalized to the cell density. The SV2-S binding was further normalized to the wells without inhibitors. The experiments were repeated at least three times. Data are presented as mean ± SEM. **(C)** SV2-S binding on MLEC surface. The tested HS mutant MLEC lines include wildtype (WT) control, *Hs2st1*^− /−^ (lack of 2S), *Hs6st1*^− /−^ (reduced 6S), *Hs6st2*^− /−^ (normal 6S), *Hs6st1^− /−^ ;2*^− /−^ (no 6S), *Sulf1^− /−^ ;2*^− /−^ (increased 6S), *Hs3st1*^− /−^ (reduced 3S), *Hs3st4*^− /−^ (reduced 3S), and *Hs3st1^− /−^ ;4*^− /−^ (greatly reduced 3S). Cell surface SV2-S protein binding was assessed by cell-based ELISA after incubation of fixed cells with his-tagged SV2-S and normalization to the cell density. The experiments were repeated 6- to 12- times and the unpaired t-test was performed for comparison between the wildtype and the mutants. Data are presented as mean ± SD. Ns, not significant; *p < 0.05, **p < 0.01.

Heparin has been reported to bind SV2-S, but the related structural information remains largely unknown. Heparin possesses *N*-sulfation (NS), 2-*O*-sulfation (2S), 3-*O*-sulfation (3S), and 6-*O*-sulfation (6S) modifications, which form binding-sites for protein ligands (Wang et al., 2002; Zong et al., 2016, 2017). To determine the type of the sulfation modification that is required for heparin to bind SV2-S, we assessed the binding of SV2-S to confluent A549 cells in the presence of heparin, or chemically modified heparins including NDS-HP, 2DS-HP, 6DS-HP, or OS-HP. Heparin showed a strong inhibitory activity with an IC50 of 9.1 ± 2.1 nM and 2DS-HP showed a comparable inhibitory activity with an IC 50 of 2.0 ± 28.7 nM (Figure 3A). The NDS-HP and 6DS-HP showed much weaker inhibitory activities with IC50 values at 3828 ± 16.3 nM and 489.8 ± 2.1 nM, respectively (Figure 3A). The OS-HP showed higher inhibitory activity than heparin (Figure 3B), 118.9 ± 2.9 nM for heparin and 53.7 ± 2.7 nM for OS-HP in new sets of the experiment. These results indicated that heparin requires its NS and 6S, but not 2S, to bind SV2-S, and that binding also depends on the overall sulfation level.

### 2-, 6- and 3-*O*-Sulfation Are Not Required for Cell Surface HS to Bind SV2-S, and the Cell Surface HS to Bind SV2-S Is Mainly Determined by Its Overall Sulfation

We examined serial HS mutant MLEC lines which were generated in our lab (Wijelath et al., 2010; Qiu et al., 2018), including the cells that are deficient in *HS 2-O-sulfotransferase* (*Hs2st^–/–^*), *HS 6-O-sulfotransferase-1* (*Hs6st1^–/–^*), *Hs6st2^–/–^*, both *Hs6st1* and *Hs6st2* (*Hs6st1^–/–^;2^–/–^*), both *HS 6-O-endosulfatase-1 and HS 6-O-endosulfatase-2* (*Sulf1^–/–^;2^–/–^*), *3-O-sulfotransferase-1* (*Hs3st1^–/–^*), *Hs3st4^–/–^*, or both *Hs3st1* and *Hs3st4* (*Hs3st1^–/–^;4^–/–^*) to extend the biochemical heparin-SV2-S binding studies and to determine the sulfation modification required for cell surface HS to bind SV2-S (Figure 3C). The cell surface SV2-S binding was assessed using a cell-ELISA approach (Wang et al., 2002; Qiu et al., 2018). *Hs2st^–/–^* MLEC HS completely lacks 2S with a slight increase of overall sulfation (Qiu et al., 2018). The *Hs2st^–/–^* MLECs showed a cell surface SV2-S binding comparable to wildtype control, indicating 2S is not required for cell surface HS to bind SV2-S. HS 6S is co-determined by Hs6sts and Sulfs. The *Hs6st1^–/–^; 2^–/–^* MLEC HS completely lacks 6S with normal overall sulfation (Qiu et al., 2018) and showed cell surface SV2-S binding comparable to wildtype control, indicating 6S is not essential for cell surface HS to bind SV2-S (Figure 3C). Intriguingly, the *Hs6st1^–/–^* and *Hs6st2^–/–^* MLECs both exhibited increased cell surface SV2-S binding (Figure 3C). The *Hs6st1^–/–^* and *Hs6st2^–/–^* MLEC HS possess a reduced and normal level of 6S, respectively, with normal overall sulfation (Qiu et al., 2018), indicating the increased cell surface SV2-S binding was due to alteration of HS fine structure, rather than overall sulfation, emerging fine structure contributes to HS binding to SV2-S. To test if increasing 6S would also alter cell surface SV2-S binding, we examined *Sulf1^–/–^;2^–/–^* MLECs which possesses increased 6S with compensatory reductions in NS and 2S and normal overall sulfation (Qiu et al., 2018). The *Sulf1^–/–^;2^–/–^* MLECs exhibited decreased cell surface SV2-S binding (Figure 3C) and illustrated alteration of HS structure, rather than overall sulfation, reduced cell surface SV2-S binding, alternatively supporting fine structure contributes to HS binding of SV2-S. 3S is the last sulfation modification in HS biosynthesis and rare in mature HS (Thacker et al., 2014; Zhao et al., 2020). The *Hs3st1^–/–^* and *Hs3st4^–/–^* MLECs both showed reduced binding of antithrombin (AT), a ligand that strictly requires 3S for binding (Qiu et al., 2018). The *Hs3st1^–/–^;4^–/–^* MLECs have further reduced AT binding (Qiu et al., 2018). The reduction of cell surface AT binding reflects that 3S is reduced in *Hs3st1^–/–^*, *Hs3st4^–/–^*, and *Hs3st1^–/–^;4^–/–^* MLEC HS. The HS on *Hs3st4^–/–^* MLECs, but not *Hs3st1^–/–^* and *Hs3st1^–/–^;4^–/–^* MLECs, has slightly reduced overall sulfation (Qiu et al., 2018). The *Hs3st1^–/–^*, *Hs3st4^–/–^* and *Hs3st1^–/–^;4^–/–^* MLECs had cell surface SV2-S binding comparable to wildtype control, showing that 3S is not required for cell surface HS to bind SV2-S (Figure 3C). In summary, these HS mutant cell studies revealed that individual *O*-sulfation type is not essential for HS to bind SV2-S on cell surface, although fine structure contributes, supporting that overall sulfation is more important for cell surface HS to bind SV2-S.

### The Entry of SARS-Cov-2 Pseudotyped VSV Into 293T-ACE2 Cells Depends on Cell Surface HS and Can Be Inhibited by HS Antagonist and Chemically Modified Heparins

**FIGURE 4:**
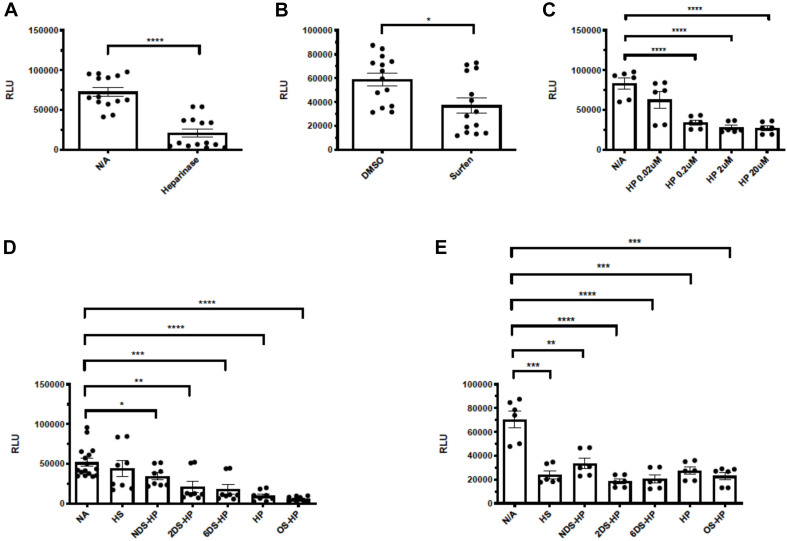
Heparinases, surfen, heparin, and chemically modified non-anticoagulant heparins block SV2-S pseudotyped virus infection of 293T-ACE2 cells. The 293T-ACE2 cells were infected with SV2-S pseudotyped virus expressing luciferase after the cells were treated with HSase (5 mU/ml) **(A)** or in the presence of surfen (20 μM) **(B)**, heparin (HP, **C**), HS or chemically-modified heparins at 2- and 20 μM **(D,E)**. The luciferase activity was measured 48 h after the virus infection. VSV pseudotyped virus was used as a control to make sure each well has comparable cell numbers (data are not shown). The experiments were repeated at least 3 times and an unpaired *t*-test was performed for the two-group comparison. Data are presented as mean ± SD. *p < 0.05, **p < 0.01, ***p < 0.001, ****p < 0.0001. RLU, relative light unit.

Our tested cell lines A549, mTHP-1, 293T, and Calu-3 express low levels of ACE2 on the cell surface and showed little to no infection by SV2-S pseudotyped VSV. Thus, they could not be used to asses the overall role of HS in SARS-CoV-2 infection. Alternatively, we generated a 293T-ACE2 cell line which stably expresses an increased, albeit low, level of ACE2 expression and could be effectively infected by SV2-S pseudotyped virus. We assessed entry of SV2-S pseudotyped virus into the 293T-ACE2 cells after treatment with HSase to degrade cell surface HS. HSase treatment reduced more than 72% SARS-Cov-2 pseudovirus entry into 293T-ACE2 cells, showing that binding of SV2-S to cell surface HS plays a key role in mediating the virus infection of host cells (Figure 4A). The 293T-ACE2 cells were inoculated with SV-2S pseudovirus in the presence of surfen [a small molecule antagonist of HS (Schuksz et al., 2008)], heparin, or chemically modified non-anticoagulant heparins including NDS-HP, 2DS-HP, 6DS-HP, and OS-HP to test if blocking the interaction of cell surface HS with SV2-S inhibited pseudovirus infection, Surfen at 20 μM inhibited SARS-Cov-2 pseudovirus entry by 52% (Figure 4B). Heparin at low concentrations (starting at 0.02 μM) showed a potent, concentration-dependent inhibition with a maximal inhibition (67%) at >2 μM (Figure 4C). At 2 μM, the de-sulfated heparins showed weaker but significant inhibitory activities with the following order: heparin > 6DS-HP = 2DS-HP > NDS-HP, and OS-HP showed a higher inhibitory activity than heparin (Figure 4D). At 20 μM concentration, heparin, HS, the desulfated heparins, and OS-HS showed comparable inhibition of SARS-Cov-2 pseudovirus infection (Figure 4E).

### G614 Mutation Increases SV2-S Binding Affinity to Heparin, and the Entry of SARS-CoV-2 G614 Mutant Into Host Cells Depends on HS and Can Be Inhibited by Heparin

**FIGURE 5:**
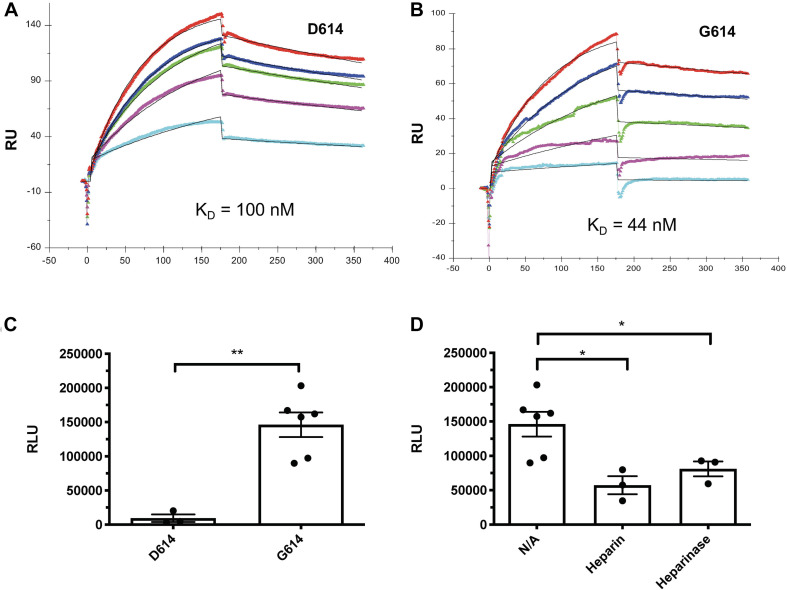
The SV2-S variant G614 protein has a higher binding affinity to heparin than the wildtype SV2-S D614 protein, and the entry of SAS-Cov-2 G614 pseudotyped virus into host cells depends on cell surface HS and can be inhibited by heparin. **(A,B)** SPR binding kinetics sensorgrams between heparin and SV2-S D614 or SV2-S G614. Concentrations of D614 were (from top to bottom): 1,000, 800, 600, 400, and 200 nM, respectively **(A)**, and G614 were (from top to bottom): 1,000, 600, 400, 200, and 100 nM, respectively **(B)**. The black curves are the fitting curves using a 1:1 Langmuir model from BIAevaluate 4.0.1. **(C)** Infection of 293T-ACE2 cells by the same titer of SARS-Cov2-D614 and SARS-Cov2-G614 pseudovirus. **(D)** SARS-Cov2-G614 pseudovirus depends on HS to infect host cells. The 293T-ACE2 cells were infected with SARS-Cov2-G614 pseudovirus expressing luciferase after the cells were treated with heparinases I-III (5 mU/ml) or in the presence of heparin (2 μM). The luciferase activity was measured 48 h after the virus infection. VSV pseudotyped virus was used as a control to make sure each well has comparable cell numbers (data are not shown). The experiments were repeated at least 3 times and an unpaired t-test was performed for two-group comparison. Data are presented as mean ± SD. *p < 0.05, **p < 0.01.

The SARS-CoV-2-S G614 variant mutated D614 into G614 and has predominated globally (Korber et al., 2020; Martin et al., 2020). A recent study reported by Zhang et al. (2020a) uncovered that the G614 mutation increased 9-fold infection of the SARS-CoV-2. Currently, the molecular mechanism underlying the increased infectivity of the mutant remains incompletely understood. We tested if the mutation alters the interaction of SV2-S with heparin by determining the kinetic binding of SV2-S G614 mutant to immobilized heparin in SPR analysis. The sensorgrams of various concentrations of SV2-S were fit globally to obtain association rate constant (*K*_*a*_), dissociation rate constant (*K*_*d*_) and equilibrium dissociation constant (*K*_*D*_) (Table 2) using the BiaEvaluation software and assuming a 1:1 Langmuir model. The wildtype D614 SV2-S and mutant G614 had binding affinity *K*_*D*_ values of 100 and 44 nM, respectively, indicating the G614 mutation strengthened the binding of SV2-S to heparin (Figures 5A,B). In addition, the SV2-S G614-pseudotyped virus showed ∼15-fold higher infection of the 293T-ACE2 cells than the SV2-S D614-pseudotyped virus (Figure 5C), similar to the recent report in literature (Zhang et al., 2020a). Pre-treatment of 293T-ACE2 cells with HSase or infection in the presence of 2 μM heparin both inhibited the SV2-S G614 pseudovirus entry into the 293T-ACE2 cells, showing that the infection by the SV2-S G614 mutant depends on host cell surface HS and can be inhibited by heparin (Figure 5D).

**TABLE 2:** Summary of kinetic data of wild type spike and mutant spike protein-heparin interactions*.

Interaction	*k*_*a*_ (1/MS)	*k*_*d*_ (1/S)	*K*_*D*_ (M)
**SV2-S D614**	1.3 × 10^4^ ± 132	1.3 × 10*^–^*^3^ ± 1.6 × 10*^–^*^5^	1.0 × 10*^–^*^7^
**SV2-S G614**	1.2 × 10^4^ ± 374	5.3 × 10*^–^*^4^ ± 3.2 × 10*^–^*^5^	4.4 × 10*^–^*^8^

*The data with (±) in parentheses are the standard deviations (SD) from global fitting of five injections.

### Dysregulated HS Expression May Contribute to the Increased Vulnerability of Lung Cancer Patients to SARS-Cov-2 Infection

**FIGURE 6:**
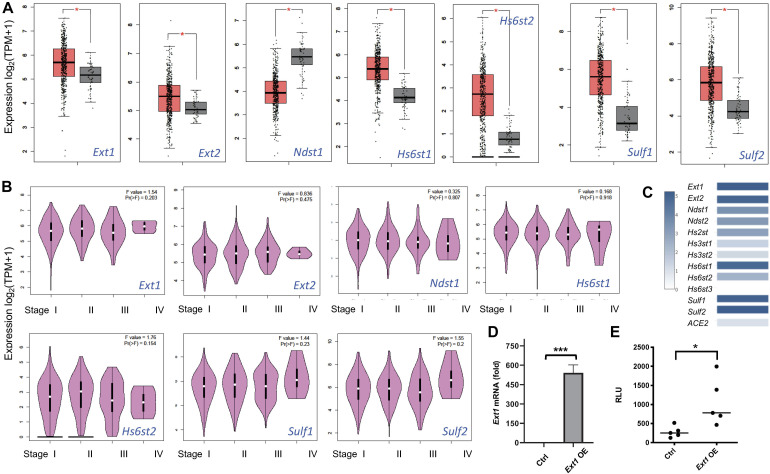
Heparan sulfate gene expression is dysregulated in lung squamous cell carcinoma and replicating the upregulated *Ext1* expression enhances SARS-Cov2 pseudovirus infection. **(A)** Dysregulated HS gene expression in lung squamous cell carcinoma (LUSC). Using Gene Expression Profiling Interactive Analysis 2 (GEPIA2) software, the data deposited in The Cancer Genome Atlas (TCGA) including 486 LUSC patients with 50 normal controls were analyzed. The orange box indicates the tumor samples while the gray one represents the normal controls. The major HS biosynthetic genes, such as *Ext1* and *Ext2* are upregulated in LUSC. *p < 0.01. **(B)** HS gene expression did not differ among different pathological LUSC stages. **(C)** The relative expression of *ACE2* and HS genes in the LUSC. **(D,E)** Overexpression (OE) of *Ext1* increased SARS-CoV2 pseudovirus infection. Forty-eight hours after infection with a human *Ext1* expression lentiviral vector, the 293T-ACE2 showed increased Ext1 mRNA expression **(D)** and were inoculated with luciferase-expressing SARS-Cov2 pseudovirus and examined for luciferase activity 48 h after the virus inoculation **(E)**. VSV pseudotyped virus was used as a control to make sure each well has comparable cell numbers (data are not shown). The experiments were repeated at least 3 times and an unpaired *t*-test was performed for two-group comparison. Data are presented as mean ± SD. *p < 0.05, ***p < 0.0001.

Several retrospective cohort studies revealed that cancer patients are at increased risk from COVID-19 severity and fatality (Liang et al., 2020; Mehta et al., 2020; Poortmans et al., 2020; Rogado et al., 2020; Yu et al., 2020; Zhang et al., 2020b). Among the major cancer types analyzed, lung cancer patients with COVID-19 have the highest fatality (Liang et al., 2020; Mehta et al., 2020; Poortmans et al., 2020; Rogado et al., 2020; Yu et al., 2020; Zhang et al., 2020b). The aggravating factor that contributes to the higher fatality of lung cancer is unknown. Using GEPIA2 (Tang et al., 2017), we analyzed the lung cancer mRNA transcript data deposited in TCGA including 486 LUSC patients with 50 normal controls. The expression of HS biosynthetic genes, including *Ext1*, *Ext2*, *Hs6st1*, *Hs6st2*, *Sulf1*, and *Sulf2* are upregulated whereas *Ndst1* is downregulated (Figure 6A), and are not different among pathological stages of LUSC (Stage I-IV) (Figure 6B). In comparison, the expression of major HS genes, including *Ext1-2*, *Ndst1-2*, *Hs2st1*, *Hs6st1-2*, and *Sulf1-2*, are much more abundant than *ACE2* (Figure 6C), suggesting that ACE2 expression is low in LUSCs compared to HS. Therefore, HS may serve as the major binding site for SV2-S to mediate SARS-CoV-2-S infection of LUSCs. In HS biosynthesis, Ext1 and Ext2 form functional heterodimers to co-polymerize HS disaccharide backbone and determine the HS chain length (expression level) in general (Kraushaar et al., 2010, 2012; Rai et al., 2020). We overexpressed *Ext1* in 293T-ACE2 cells to assess the functional consequence of upregulated *Ext1* expression in the vulnerability of lung cancer to SARS-CoV-2 (Figure 6D). The *Ext1* overexpressing 293T-ACE2 cells showed higher infection of SV-2S pseudovirus than the scrambled control (Figure 6E), suggesting the *Ext1* upregulation may be one of the mechanisms underlying the higher susceptibility of lung cancer patients to SARS-CoV-2 infection.

## Discussion

In this study, we assessed 9 cell types derived from different tissues, including epithelial, neuronal, macrophage, kidney and endothelial cells to determine the relative importance of HS and ACE2 in cell surface SV2-S binding. Four of the cell lines (A549, 293T, mTHP-1, and Calu-3) express low levels of ACE2. We observed that cell surface SV2-S binding depends on and highly correlates with HS expression. The cell surface SV2-S binding on ACE2-expressing cell lines also correlates with ACE2 level, but with a lower correlation coefficient than HS. More importantly, the correlation between cell surface SV2-S binding and ACE2 expression was diminished when the cells were pretreated with HSase to remove cell surface HS. These observations indicate that cell surface HS, not ACE2, mediates in the initial phase of cell surface binding of SV2-S. We also observed that HSase treatment greatly inhibited SV2-S pseudotyped virus infection of 293T-ACE2 cells, indicating that cell surface HS is essential for SARS-CoV-2 infection. Intriguingly, when we tested with high ACE2-expressing 293T cells, the dependence of cell surface HS for SV2-S binding was diminished. One explanation for this discrepancy is HS acts as a co-recptor in SV2-S-ACE2 signaling, as suggested recently (Clausen et al., 2020). Low expression of ACE2 on the cell surface represents the limiting factor for SV2-S-ACE2 signaling and requires the abundantly-expressed HS to act as a co-receptor to enrich and facilitate SV2-S binding to ACE2. However, when ACE2 expression is high, the HS co-receptor function is dispensable. Some studies have reported that interferon-mediated induction of ACE2 expression occurs upon SARS-CoV-2 infection (Fu et al., 2020; Su and Jiang, 2020; Tan et al., 2020), suggesting that blocking HS-SV2-S binding by heparin may not be sufficient (Efendizade et al., 2020; Hippensteel et al., 2020; Menezes-Rodrigues et al., 2020) and would need to be combined with other mechanism-based agents to most effectively to treat and/or prevent COVID-19.

In previous studies, we determined that monomeric SV2-S-Fc exhibited an exceptionally high-affinity binding to heparin in SPR analysis with a *K*_*D*_ = 40 pM (Kim et al., 2020). In current SPR analysis, we tested monomeric His-tagged SV2-S from the same vendor and determined that the His-tagged SV2-S remained high-affinity binding to heparin too, but the *K*_*D*_ was reduced to 100 nM. The difference in binding affinity may be due to the Fc-tagged SV2-S is a disulfide-bonded dimer and contains two SV2-S monomer whereas His-tagged SV2-S is a monomer. Meanwhile, SV2-S contains extensive *N*- and *O*-glycosylation (Shajahan et al., 2020; Wang et al., 2020; Watanabe et al., 2020; Xu et al., 2020) and the glycosylation may play a role in protein folding thereby affects the interaction of SV2-S with heparin. It is possible the difference in binding affinity might also be contributed by their glycosylation difference between SV2-S-Fc and His-tagged SV2-S; however, this needs to be experimentally determined.

We examined SV2-S binding to serial chemically modified heparins and *O*-sulfation deficient HS mutant MLEC lines to determine if specific modifications are required for HS to mediate cell surface SV2-S binding. We observed both NS and 6S, compared to 2S, are more important for heparin to bind His-tagged SV2-S and the overall sulfation, instead of the individual sulfation modification type, plays the major role in determining the binding affinity of heparin to SV2-S. In agreement with 2DS-heparin study observation, *Hs2st^–/–^* MLECs showed normal cell surface SV2-S binding although *Hs2st^–/–^* MLEC HS completely lacks 2S and has a slight increase of overall sulfation, indicating that 2S is not required for HS to bind SV2-S on cell surface.

The consequences of manipulating 6S in HS-mediated SV2-S binding on MLEC surface appears complex. Although 6DS-heparin displayed a reduced binding affinity to SV2-S, the *Hs6st1^–/–^;2^–/–^* MLECs showed a normal cell surface SV2-S binding. The *Hs6st1^–/–^;2^–/–^* MLEC HS does not have 6S with compensatory increases of NS and 2S which result in an unchanged overall sulfation (Qiu et al., 2018), the normal SV2-S binding on *Hs6st1^–/–^;2^–/–^* MLEC cell surface led to our conclusion that 6S is not essential for HS to bind SV2-S and overall sulfation is more important. A recent study reported by Clausen et al. (2020) showing the binding of SV2-S on *Hs6st1^–/–^;2^–/–^* Hep3B cells is significantly reduced. This reduced cell surface SV2-S binding is most likely due to decreased overall sulfation modification since the *Hs6st1^–/–^;2^–/–^* Hep3B HS has reduced 6S and a significantly reduced overall sulfation (Anower et al., 2019).

the *Hs6st1^–/–^* and *Hs6st2^–/–^* MLECs showed increased cell surface SV2-S binding. Since the *Hs6st1* deletion reduces 6S but does not alter NS, 2S and overall sulfation, and the *Hs6st2* deletion does not change NS, 2S, 6S and overall sulfation (Qiu et al., 2018), we therefore concluded that the increased cell surface SV2-S binding is due to the *Hs6st* deletion generates more SV2-S binding site and/or higher binding affinity, and fine structure contributes to HS to bind SV2-S. This notion is alternatively supported by study of *Sul1^–/–^;2^–/–^* MLECs which showed reduced cell surface SV2-S binding. The *Sul1^–/–^;2^–/–^* MLEC HS possesses increased 6S with compensatory decreases of NS and 2S and no change of overall sulfation (Qiu et al., 2018), showing the decreased SV2-S binding on the *Sul1^–/–^;2^–/–^* MLECs is not related to overall sulfation but due to decreasing SV2-S binding site and/or lowering binding affinity. Our study of *Hs6st-* and *Sulf-*deficient MLECs concluded that fine structure contributes also to HS binding of SV2-S.

We examined *Hs3st1^–/–^*, *Hs3st4^–/–^* and *Hs3st1^–/–^;4^–/–^* MLEC cell lines, which have normal NS, 2S, and 6S with reduced AT binding, indicating 3S is reduced or diminished to determine if 3S contributes to HS-mediated SV2-S binding on cell surface (Qiu et al., 2018). The *Hs3st4^–/–^* MLEC HS, not *Hs3st1^–/–^* and *Hs3st1^–/–^;4^–/–^* MLEC HS, has slightly reduced overall sulfation, supporting the notion that 3S is a type of rare modification within HS (Thacker et al., 2014). The *Hs3st4^–/–^* MLECs, *Hs3st1^–/–^* and *Hs3st1^–/–^;4^–/–^* MLECs all showed normal levels of cell surface SV2-S binding, indicating 3S is not essential for HS to bind SV2-S and supporting that overall sulfation plays a major role in mediating this interaction.

Recently, Tiwari et al. (2020b) reported that overexpression of *Hs3st3b* preferentially increased SV2-S-mediated cell-to-cell fusion. In mammals, the seven isozymes of *Hs3st* are divided into two subgroups based on the homology of the sulfotransferase domain: the “AT-type” which includes *Hs3st1* and *Hs3st5*, and the “gD-type” that contains *Hs3st2*, *Hs3st3a*, *Hs3st3b*, *Hs3st4*, and *Hs3st6* (Lawrence et al., 2007; Liu and Pedersen, 2007). The “AT-type” and “gD-type” Hs3sts generate differently structured 3S-modified ligand binding sites for antithrombin and glycoprotein gD of Herpes simplex virus-1, respectively (Lawrence et al., 2007). We recently reported that deletion of “AT-type” *Hs3st1*, “gD -type” *Hs3st4*, or both reduced MLEC surface AT binding (Qiu et al., 2018); therefore, we postulate the increased SV2-S-mediated cell-to-cell fusion by *Hs3st3b* overexpression is most likely due to an increase of HS overall sulfation.

As shown in this study, and reports from our own and other groups, heparin potently inhibited SV2-S-mediated pseudovirus entry into host cells (Clausen et al., 2020; Tandon et al., 2020). We and others also reported that low molecular weight heparin and chemically-split heparin have strong inhibitory effects too (Clausen et al., 2020; Tandon et al., 2020). Extending these reports, we examined surfen, a small molecule HS antagonist (Schuksz et al., 2008), and several chemically modified, non-anticoagulant heparins, which showed anti-inflammatory effects in our previous studies (Wang et al., 2002; Zhang et al., 2012). We observed that surfen and chemically-modified non-anticoagulant heparins including NDS-HP, 2DS-HP, 6DS-HP, and OS-HP all inhibited SV2-S-mediated pseudovirus entry into host cells. Surfen has been used safely in patients for a long time (Schuksz et al., 2008) and heparin is a commonly used anticoagulant in patients; therefore, surfen and the derivatives of heparin, the chemically-modified heparins, may be applied safely to prevent or treat COVID19. Importantly, the chemically-modified heparins do not have anticoagulant activity, avoiding the potential bleeding side effect of heparin treatment.

SARS-CoV-2 variants with SV2-S G614 mutation now predominate globally (Walls et al., 2020b; Zhou et al., 2020). A recent study observed that the SV2-S G614 pseudotyped virus enters ACE2-expression cells more efficiently than the SV2-S D614 wildtype pseudotyped virus (Zhang et al., 2020a). The increased infectivity of the SV2-S G614 pseudotyped virus correlates with less S1-domain shedding and higher SV2-S incorporation into the virion. The G614 mutation does not affect SV2-S binding to ACE2 or neutralization sensitivity of the pseudovirus, suggesting that G614 mutation may assemble more functional SV2-S into the virion to increase infectivity (Zhang et al., 2020a). In the current study, we found that the G614 mutation strengthens SV2-S binding to heparin, suggesting that the G614 mutation may enhance SV2-S interaction with host cell surface HS thereby increase infectivity of the mutant virus. This observation reveals a potential novel mechanism underlying the higher infectivity of the SV2-S G614 variant. More importantly, the entry of the SV2-S G614 pseudotyped virus into host cells depends on host cell surface HS and can be inhibited by heparin, suggesting heparin, surfen, and chemically-modified heparins may be effective to prevent or treat SV2-S G614 variant infection too.

The severity of COVID-19 and the course of the infection is heterogeneous and appears to be more severe in the elderly and individuals with underlying comorbidities. COVID-19 does not appear to significantly impact children, a pattern atypical for most viral respiratory diseases. Several retrospective cohort studies have emerged that cancer patients are at increased risk of COVID-19 severity and fatality due to underlying malignancy, treatment-related immunosuppression, or increased comorbidities (Liang et al., 2020; Mehta et al., 2020; Poortmans et al., 2020; Rogado et al., 2020; Yu et al., 2020; Zhang et al., 2020b). Among the major cancer types analyzed, lung cancer patients show the highest fatality rates for COVID-19. A recent comprehensive clinical study identified a total of 218 COVID-19 positive patients with a malignant diagnosis (Mehta et al., 2020). In this report, a total of 61 (28%) cancer patients died from COVID-19 emerging lung cancer patients being the most vulnerable population, with a fatality rate of 55%. Currently, the mechanisms underlying the higher vulnerability of cancer patients to SARS-Cov-2 infection remain largely unknown (Liang et al., 2020; Mehta et al., 2020; Poortmans et al., 2020; Rogado et al., 2020; Yu et al., 2020; Zhang et al., 2020b). In this study, we uncovered that the expression of several HS biosynthetic genes, including *Ext1*, is upregulated in lung cancer. Replicating the upregulated *Ext1* expression increased infection of the host cells by SARS-CoV-2 pseudovirus, and the increased infection can be blocked by HSase and heparin, suggesting heparin and its derivatives, as well as surfen, may be effective to prevent or block SARS-CoV-2 infection in cancer patients.

## Conclusion

Our studies demonstrated that host cell surface SV2-S binding and SV2-S-mediated SARS-CoV-2 pseudovirus infection depends on host cell surface HS and can be blocked by heparin lyase, HS antagonist surfen, heparin, and non-anticoagulant heparin derivatives. HS binding to SV2-S and the inhibitory effects of heparin and heparin derivatives are mainly determined by their overall sulfation instead of a specific type of sulfation. This conclusion is supported by mutant cell studies, which determined that 2S, 6S, and 3S are not essential for HS to bind SV2-S on cell surface. Our HS mutant cell study also emerged that the binding of HS to SV2-S is contributed by its fine structure too, although this appears minor comparing to its overall sulfation. Our studies also determined that the G614 mutation strengthens binding of SV2-S to heparin, suggesting the increased SV2-S-HS interaction may be one of the mechanisms underlying the higher infectivity of the SARS-CoV-2 G614 variant. Furthermore, we showed HS expression is upregulated in lung cancer and higher HS expression increased host cell infection of SARS-CoV-2, delineating a mechanism accounting for the high vulnerability of lung cancer to SARS-CoV-2 infection. Lastly, we demonstrated that heparin lyase and heparin effectively blocked host cell infection by SARS-CoV-2 G614 variant pseudovirus and increased infection caused by upregulated HS expression, suggesting that heparin, heparin derivatives, heparin lyase, and surfen may be applied to prevent or treat SARS-CoV-2 G614 variant infection and infection of cancer patients.

## Data Availability Statement

Publicly available datasets were analyzed in this study. This data can be found here: TCGA-LUSC data was downloaded from NIH CDC Data Portal: https://portal.gdc.cancer.gov/projects/TCGA-LUSC, accession number: dbGaP Study Accession phs000178.

## Author Contributions

JY, WJ, HY, JF, XS, and HQ conducted the experiments, acquisition, and analysis of the results. JY, FZ, MT, PA, RL, and LW designed the experiments and wrote the manuscript. All authors reviewed the results and approved the final version of the manuscript.

## Conflict of Interest

The authors declare that the research was conducted in the absence of any commercial or financial relationships that could be construed as a potential conflict of interest.

## Abbreviations

2S: 2-*O*-sulfation
3S: 3-*O*-sulfation
6S: 6-*O*-sulfation
2DS-HP: 2-*O*-desulfated heparin
6DS-HP: 6-*O*-desulfated heparin
ACE2: angiotensin-converting enzyme 2
Ext1: exostosin-1
Ext2: exostosin-2
GEPIA2: gene expression profiling interactive analysis 2
HLMVEC: human lung microvascular endothelial cell
HS: Heparan sulfate
Hs2st: heparan sulfate 2-*O*-sulfotransferase
Hs3st1: heparan sulfate 3-*O*-sulfotransferase-1
Hs3st4: heparan sulfate 3-*O*-sulfotransferase-4
Hs6st1: heparan sulfate 6-*O*-sulfotransferase-1
Hs6st2: heparan sulfate 6-*O*-sulfotransferase-2
HSase: heparinases I–III
LUSC: lung squamous cell carcinoma
MLEC: mouse lung endothelial cell
mTHP-1: macrophage induced from THP-1
NDS-HP: *N*-desulfated /*N*-acetylated heparin
NS: *N*-sulfation
OS-HP: oversulfated heparin
PMA: phorbol 12-myristate-13-acetate
RT: room temperature
SARS: severe acute respiratory syndrome
SARS-CoV-2: severe acute respiratory syndrome coronavirus 2
SPR: surface plasmon resonance
Sulf1: heparan sulfate 6-*O*-endosulfatase-1
Sulf2: heparan sulfate 6-*O*-endosulfatase-2
SV2-S: SARS-CoV-2 spike protein
TCGA: The Cancer Genome Atlas.
